# Perspectives for the allergenicity assessment of key allergens in GM plants

**DOI:** 10.1186/2045-7022-3-S3-P88

**Published:** 2013-07-25

**Authors:** A Fernandez, C Mills, M Lovik, A Spoek, A Germini, A Mikalsen, J-M Wal

**Affiliations:** 1 European Food Safety Authority, Parma, Italy; 2 Universitiy of Manchester, Manchester, UK; 3 Norwegian Institute of Public Health, Oslo, Norway; 4 Inter-University Research Centre for Technology, Work and Culture, Graz, Austria; 5 Norwegian Scientific Committee for Food Safety, Oslo, Norway; 6 INRA-CEA, Gif sur Yvette Cedex, France

## Background

According to EU legislation, genetically modified plants (GM plants) should follow a scientific assessment of any risk that they may pose to human and animal health, including an allergenicity assessment. According to the EFSA guidance^1^, the allergenicity assessment of GM plants is structured into the assessment of i) the newly expressed proteins and ii) the whole GM plant. For the latter and as a part of the assessment, if the GM plant receiving the introduced gene(s) is known to be allergenic, its endogenous allergenicity is compared with that of its non-GM comparator(s).

## Methods

Historically, the assessment of endogenous allergenicity has been performed by analytical methodologies based on the use of human sera. Currently, new analytical methods and profiling techniques (*e.g.* mass spectrometry approaches) not based on human sera are now evolved and could be used for these purposes. In view of these new developments, EFSA recommended the possible inclusion of key allergens in the comparative compositional analysis to limit the use of human sera [1,2].

## Results

There are several limitations associated with the use of human sera, *e.g.* availability, reproducibility, variability from allergic individuals, difficulty of obtaining well-characterised allergic individuals and sera, etc. New technologies, which do not require human sera for the identification and quantification of allergens, have shown to be very useful for risk assessment [3-5]. They may provide important and reliable information on the potential for unintended effects on the endogenous allergenicity (*i.e.* over expression of allergens) due to the genetic modification.

## Conclusion

In order to avoid the use of human sera, alternative methods based on recent technological advances need to be considered. For the allergenicity assessment of GM plants, the inclusion of key allergens in the comparative compositional analysis, as an additional parameter to be measured by these new methods, could provide robust and reliable information for risk assessment. In this context, when a GM plant is compared with its non-GM comparator(s), an important aspect to be considered is the natural variability and the biological relevance of the identified differences.

## Disclosure of interest

None declared.
